# Infection, Disease, and Biosocial Processes at the End of the Indus Civilization

**DOI:** 10.1371/journal.pone.0084814

**Published:** 2013-12-17

**Authors:** Gwen Robbins Schug, K. Elaine Blevins, Brett Cox, Kelsey Gray, V. Mushrif-Tripathy

**Affiliations:** 1 Department of Anthropology, Appalachian State University, Boone, North Carolina, United States of America; 2 Institute of Archaeology, University College, London, United Kingdom; 3 Department of Archaeology, Deccan College Post-Graduate Research Institute, Pune, India; University of Oxford, United Kingdom

## Abstract

In the third millennium B.C., the Indus Civilization flourished in northwest India and Pakistan. The late mature phase (2200-1900 B.C.) was characterized by long-distance exchange networks, planned urban settlements, sanitation facilities, standardized weights and measures, and a sphere of influence over 1,000,000 square kilometers of territory. Recent paleoclimate reconstructions from the Beas River Valley demonstrate hydro-climatic stress due to a weakened monsoon system may have impacted urban centers like Harappa by the end of the third millennium B.C. the impact of environmental change was compounded by concurrent disruptions to the regional interaction sphere. Climate, economic, and social changes contributed to the disintegration of this civilization after 1900 B.C. We assess evidence for paleopathology to infer the biological consequences of climate change and socio-economic disruption in the post-urban period at Harappa, one of the largest urban centers in the Indus Civilization. Bioarchaeological evidence demonstrates the prevalence of infection and infectious disease increased through time. Furthermore, the risk for infection and disease was uneven among burial communities. Corresponding mortuary differences suggest that socially and economically marginalized communities were most vulnerable in the context of climate uncertainty at Harappa. Combined with prior evidence for increasing levels of interpersonal violence, our data support a growing pathology of power at Harappa after 2000 B.C. Observations of the intersection between climate change and social processes in proto-historic cities offer valuable lessons about vulnerability, insecurity, and the long-term consequences of short-term strategies for coping with climate change.

## Introduction

**Figure 1:**
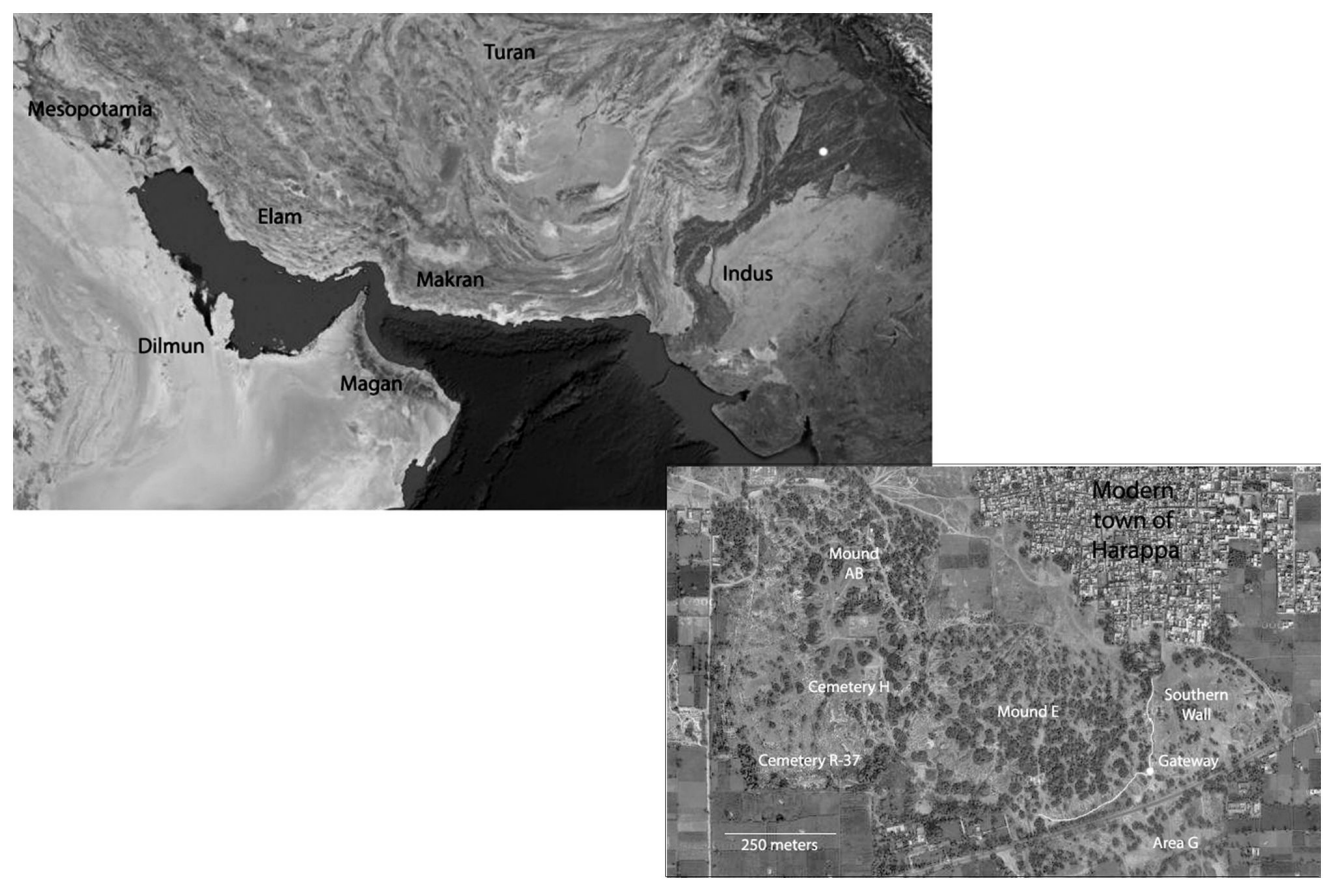
Map of the Third Millennium Interaction Sphere, the Geographic Extent of the Indus Civilization, and the location of the city of Harappa.

We examined the human skeletal remains from Harappa, one of the largest cities in the Indus Civilization (Figure 1), to determine the impact of climate, economic, and social changes that accompanied the disintegration of this proto-historic society. Using an analysis of paleopathology in the human skeletons from three different burial populations that span the decline of this ancient civilization, we assessed the signature of large-scale social processes (urbanization, extensive culture contact, migration, social differentiation, and the economic and social changes that led to the collapse of a relatively weak prehistoric state) in the epidemiological profile. Combined with prior evidence for inter-personal violence [1], we estimate factors that determined the relative risk of social suffering through time and discuss the implications for human security during periods of climate and culture change in this prehistoric complex society.

### The Harappan Civilization

The Indus Civilization is best known for its late mature phase (2200-1900 B.C.) when it was characterized by large, well-organized cities, sanitation facilities and sophisticated water management practices, an undeciphered script, standardized system of measurements, craft specialization, and participation in the third millennium B.C. Interaction Sphere [2–8]. The Indus civilization has also gained attention as a rare example of a heterarchical prehistoric state, which developed without strong, centralized social control or structural violence [6,9,10]. But it is perhaps best known for its ‘disintegration’ in the Late Harappan phase (1900-1300 B.C.)—retraction of the interaction sphere, depopulation of urban centers, re-organization of the human population, and re-emphasis on agrarian village life for most of the second millennium B.C. 

The Harappan Civilization developed in the context of a semi-arid climate that was pervasive in South Asia for the latter half of the Holocene [11–18]. Since it was first proposed as a factor in the demise of the Indus Civilization, debates about the role of climate and environmental changes have raged on [19–27] but it has become increasingly clear that by 2800 B.C., aridity levels in the Indus Valley were broadly similar to contemporary levels [28–32] until a period of destabilized environment—fluctuating rainfall, increased seasonality, and accelerate channel migration—began in the Indus Valley after 2000 B.C. [31,32]. From 2200-1700 B.C., a significant rapid climate change event in South Asia saw disruptions in monsoon rainfall and significant changes in fluvial dynamics along the Indus Rivers, including the Beas River [13,14,18,29,30,33–40]. 

Increasing aridity initially occurred in the context of a flourishing interaction sphere that spanned West and South Asia in the third millennium B.C. [6,41–44]. Historical records from Mesopotamia describe regular trade with ‘Meluhha’ (the Indus Valley) from 2400-2000 B.C. [43]. Harappans manufactured etched, biconical carnelian beads, shell, faience and steatite ornaments, ivory, copper, and ceramic items, cotton, silk, jute, cloth, barley, oil and other perishables [43,45–51]. Exports focused on raw materials for these products, items which have been recovered from sites around the Persian Gulf region; Harappan seals have also been recovered; and cylindrical seals resembling those from Mesopotamia have been recovered at Indus urban centers as well [3,6,43,44,48]. 

Participation in the interaction sphere facilitated a period of rapid urbanization at the city of Harappa, creating a dense and heterogeneous population in the ancient city. Cities are political, economic, and ceremonial centers that can offer opportunities unavailable in the hinterlands [44]. Technology, production, and consumption transformed Indus society, particularly in period IIIC, when population growth was at its fastest rate: high levels of immigration disrupted the formerly organized settlement pattern; houses in the core areas of the city spilled over onto the streets and ‘suburban’ areas sprang up on low mounds to the west and northwest of the city center [41,42]. 

After 1900 B.C., in the Late Harappan phase, population density was diminished and settlement focused largely in the core areas of the city. Declining sanitation conditions and an increasingly disorganized settlement plan indicate disruptions to authority were systemic [41,44,52]. Disruptions in the exchange network also occurred after 2000 B.C. [35,37,53–55] at a time when West Asian trading partners were responding to their own rapid climate change event. At this point, Magan and Dilmun are mentioned more frequently in Mesopotamian writings while references to Meluhha largely disappear and material evidence of trade interactions declines [44]. Large-scale depopulation of Indus cities in the Late Harappan phase weakened Indus society. Late Harappan settlements flourished in Gujarat and Rajasthan while only a handful of settlements remained occupied in the Beas River Valley [56–62]. 

Climate change is a significant challenge for human communities but climate alone does not determine the fate of human societies; it exists as one variable intertwined with other political, social, historical, and cultural forces [63–69]. A translation of the social forces at work in the creation of the urban environment thus serves as a context for understanding its collapse. When mortuary treatment and bioarchaeological evidence are considered, three core features of Indus society emerge—Indus cities were heterogeneous, socially differentiated, and hierarchical. As discussed above, heterogeneity in a large city is expected, particularly in the context of rapid population growth and participation in a wide reaching exchange network. A heterogeneous composition at Harappa was recently confirmed by preliminary analysis of isotopic signatures in skeletons from the urban phase cemetery R-37, some of which demonstrated a non-local origin [70]. Although the female skeletons from cemetery R-37 that were studied had isotopic signatures consistent with local origins, the sample size was small and there are probably males and females of local and non-local origins buried here. The isotopic data indicate that individuals buried in Cemetery R-37 shared some aspect of occupational, affinal, or consanguinal identity but ‘Harappans’ were diverse in regard to their geographic origins. 

Archaeologists have suggested Indus cities were governed by an elite class, or by competing elite groups based on features of the settlement organization, construction of high walls around the mounds, water management practices, and presence and patterning of artifact types in Indus cities [6,41,42,44,70–72]. This model is supported by mortuary data. Given the large population size of the city, the majority of Harappans were not interred in the city cemeteries. In addition, hundreds of people were buried in cemetery H during the post-urban period but a small contingent of human crania, 50% of which demonstrated evidence for traumatic injury, were disposed of in an ossuary outside the city [1,7,73]. Evidence for social differentiation and exclusion at Harappa indicate that even if organization on a larger scale was relatively weak, social differentiation and control were exercised locally within the city [1,10]. 

Urbanization, migration, extensive culture contact, and climate change all present significant social and biological challenges for human populations, particularly the introduction of new pathogens [74]. Both molecular and skeletal evidence suggest that *Mycobacterial tuberculosis* Complex (MTBC) lineages have been circulating in South Asian populations for millennia [75–77]. A high degree of latency in MTBC infections indicates a long-term association with human communities, near the origin of urban life [78]. *M. tuberculosis* has infected human populations in West Asia since at least 7000 B.C. [79]; tuberculosis has been present in Egypt since 3500-2650 B.C. [80]. Molecular biology studies demonstrate that polymorphisms associated with resistance to members of the MTBC lineage have been conserved in South Asian populations for millennia [75]. Thus the molecular evidence lends support to the hypothesis that MTBC lineages have been circulating in South Asia since at least the late Holocene, when maintenance in human populations would have been facilitated by significant increases in population density and culture contact [81]. Bioarchaeological research recently confirmed that leprosy was present in a rural Indus outpost in Rajasthan, India by 2000 B.C. [82] providing additional impetus to look for evidence of the disease in proto-historic, urban populations in South Asia.

In this study, we examine the hypothesis that increased population density and culture contact led to new opportunities for infection and infectious disease in South Asian populations. Based on molecular and bioarchaeological evidence, we predicted that evidence for infection and infectious disease will be present in the skeletal remains from Harappa. Furthermore, based on our previous bioarchaeological research [1], we predicted that the prevalence of infection and disease will not be evenly experienced across all segments of the population. Our previous work on skeletal material from Harappa demonstrated that violence was a part of life in the ancient city and that the prevalence of violent injury increased through time with the strains of ecological and social change in the Late Harappan period [1]. The data indicated strong differences in the risk for violent injury among different burial populations through time, suggesting that aspects of individual and community identity shaped the risk for violent injury. In that paper, we joined scholars who have argued against the long-standing view that the Indus civilization was peaceful [83] but we also hypothesized that these data could be interpreted as evidence that vulnerability in Harappan society was determined through a process of structural violence—unequal power, uneven access to resources, systematic oppression, and exploitation that kills through the denial of basic needs and outright violence. We suggested than an examination of differences among burial areas in the epidemiological profile could provide additional support for that hypothesis.

Our goal here is to assess whether there is evidence for systematic structural differences in the risk for infection and infectious disease, similar to those previously reported for interpersonal violence [1]. In complex societies, political, economic, social, and historical forces leave marginalized citizens more vulnerable to food insecurity, risk of violence, and infectious disease [84–90]. In the context of climate, social, and economic changes, these social forces and structural inequalities often become exacerbated and consequences are most acutely felt by the most vulnerable citizens [91–98]; this vulnerability is inscribed on the human skeleton [99–101].

## Materials and Methods

### The Human Skeletal Remains from Harappa

**Figure 2:**
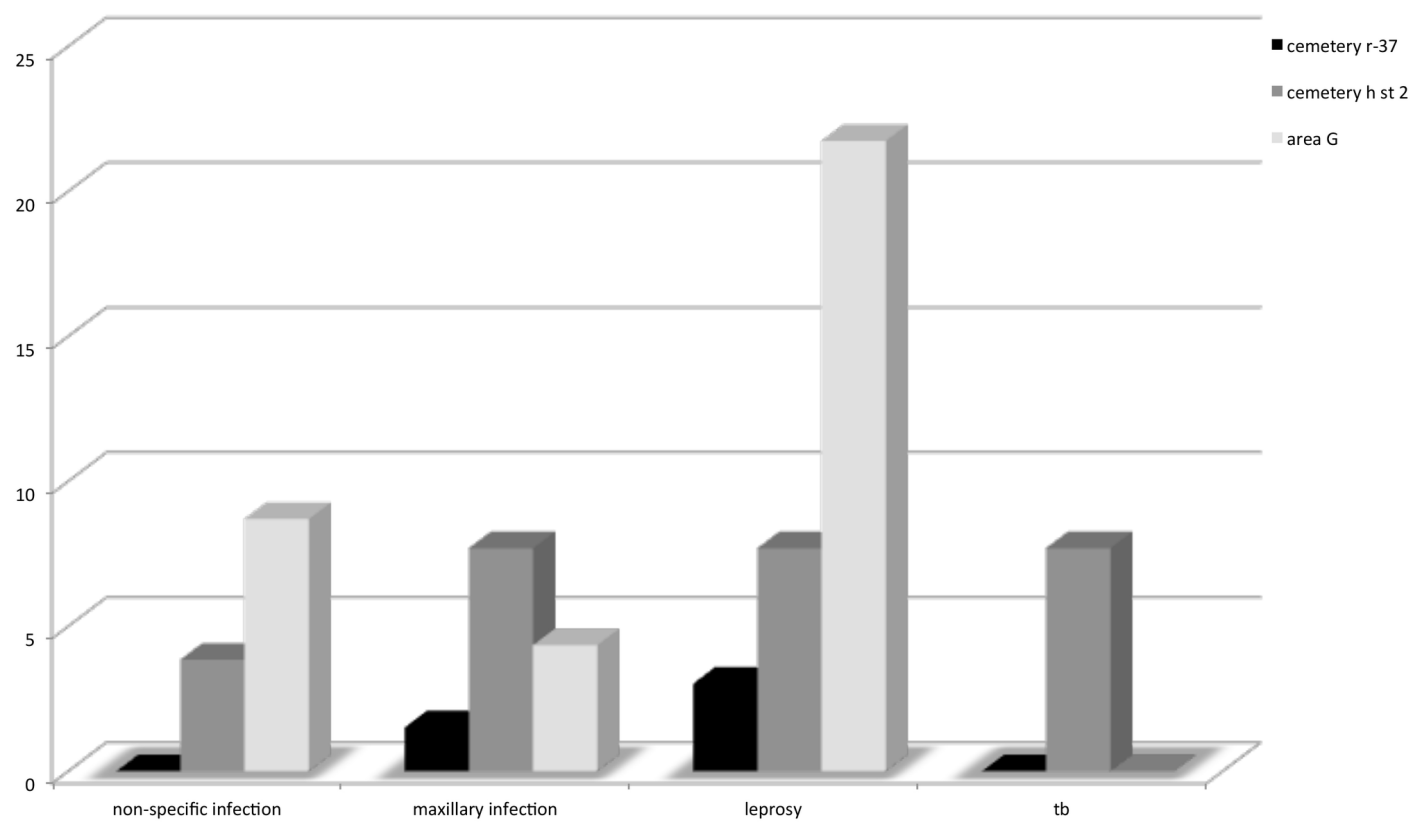
Prevalence of infection and disease in three mortuary assemblages from Harappa.

This paper presents an analysis of paleopathology across the urban to post-urban transition at Harappa. All necessary permits were obtained for the described study, which complied with all relevant regulations. The Anthropological Survey of India allowed access to the collections and the museum staff provided research support. We examined 160 individuals (67% of the total number excavated) from three main burial areas at Harappa: an urban period cemetery (R-37), a post-urban cemetery (H), and an ossuary (Area G) (Figure 2). This total represents all of the individuals excavated from 1923 to 1967 that have survived to date. Detailed scientific analyses have also been published on 90 additional individuals recovered from Cemetery R-37 by the HARP project and those remains are not included in this analysis [102–107]. Cemetery R-37 came into use in the mature period at Harappa; burials span approximately 500 years (2550-2030 B.C.) [108]. The cemetery is comprised of local and non-local individuals, a group that may represent traders, merchants or craftspeople [70]. Some of these burials were the most elaborate and the richest of any cemetery at the site, although there was considerable variation among individual graves [3,70]. In all, they contained pottery; bangles, necklaces, beads, and amulets of semi-precious stone, gold, and steatite; toilet objects such as mirrors, spoons, and small containers; and other grave goods [3,6,46,103,104,107,109]. 

Of 209 skeletons excavated from Cemetery R-37, 66 (31.6%) were available at AnSI (Anthropological Survey of India) for the present research. Of these 66, 16 were from complete burials, 29 were from fractional burials, and 21 were from multiple burials. Most of the burials were adults (64/66) but there were two immature individuals present (both children over five years of age). This paper primarily concerns an analysis of the remains that were excavated prior to independence. These remains are stored at Anthropological Survey of India, Kolkata. The preservation, completeness, morphology and metrics are provided in several books, monographs, and articles already published [73,110,111]. These remains are not the same sample described in previous studies of paleopathology; those remains were recovered during the HARP project from 1986-1989 [102–106,112]. That sample was skeletally relatively healthy and mainly suffered from a low prevalence of anemia, enamel defects, and moderate dental caries, suggesting a small amount of developmental stress and a mixed economic system with heavy reliance on agricultural effort for subsistence. 

 Cemetery H represents a late extension of Cemetery R-37 [113]. This burial ground consisted of two strata—the Late Harappan phase is represented in Stratum II (1900-1700 B.C.) and a Chalcolithic phase is represented in Stratum I (1700-1300 B.C.) . In stratum II, the dead were laid out in an extended posture and occasionally with legs somewhat flexed; they were buried with little ornamentation [8]. Stratum I skeletons were interred in funerary jars. Of the 78 individuals excavated from Cemetery H, 26 individuals from Stratum II are available for study at AnSI; 20 adults and six immature individuals. 45 individuals from Stratum I are available for study at AnSI (31 adults and 14 immature individuals) but in this paper, we are only considering burials from stratum II because our questions concern the transition from the Mature Harappan period IIIC to the Late Harappan, period IV. The Stratum I burials date to period V, a much later time period; the analysis of pathological lesions in that sample is not apropos to the current research questions and will be reported at a later date.

 Area G is located in a low-lying field outside the southeastern wall of the city, just beyond a sewage drain built under the southeastern gateway. This locality consisted of poorly preserved architectural remains and an ossuary, which contained a small number of goblets, vases, and offering dishes as well as 20 human crania, three human mandibles, five human long bones, a human scapula, and two partial human vertebral columns. A bovine cranium and a canine vertebral column were also found in the ossuary remains [7]. No absolute dates are available for Area G and the ossuary was relatively dated using the ceramic typology. Vats described the Area G ceramics as closely resembling those of the habitation area, with some resemblance in forms with Cemetery R-37 but little affinity with Cemetery H. “Hence whatever may be the interval in time between the two mounds and Cemetery H, the Area G very likely stands between the two.” (pg. 200) As Cemetery R-37 extends to 2030 B.C. and Cemetery H is dated to 1900-1300 B.C. (strata I and II), this interpretation would suggest dates of roughly 2000-1900 B.C. for Area G. A minimum number of 23 individuals (12 adults and 9 subadults) were interred here and were available for study at AnSI. 

### Bioarchaeological methods

The available skeletal material from Harappa is fragmentary and many individuals are incomplete, poorly preserved, and some elements are partially covered with a thick layer of vinyl acetate preservative, which sometimes hindered macroscopic evaluations of pathology. The preservative was removed with a soft toothbrush when possible; otherwise observations were limited to observable surfaces, where vinyl acetate had not been applied. 

 Because this skeletal collection was initially studied for evidence of ‘racial’ affinities [73], metrical analysis has been published previously [73,110] and cranial material was preserved in this collection more frequently than any other element. We estimated age-at-death for immature individuals based on dental development and eruption timing [114,115] and timing of epiphyseal fusion [116]. Sexing of subadults was not attempted. We used Standards for Data Collection to determine demographic characteristics from the adult remains [117]. Cranial elements were the most commonly preserved but yield less accurate sex estimates and less precise age estimates than other elements. Sex was estimated using pelvic indicators when the pelvis was present; otherwise cranial features were used [116]. For individuals only represented by cranial material, age was estimated using cranial suture stenosis and dental attrition [118], the latter was preferred when teeth were available. The specimens were categorized into three age categories: Young adult (18-29), Middle adult (30-54), and Older adult (55+). These categories are consistent with earlier studies of the material from Cemetery R-37 [103–105,112]. Demographic characteristics are provided in Table 1.

**Table 1:** Age and Sex of Skeletons from Harappa included in this analysis.

	**N total**	**n examined **	**Immature**	**Young adult (18-34)**		**Middle adult (35-54)**		**Old adult (55+)**		**Indet. Age (adult)**
				*male *	*female *	*male *	*female *	*male *	*female*	
*Cemetery R-37*	108	66	3	3	9	2	3	7	3	31
*Cemetery H*										
*Stratum II*	26	26	6	0	4	0	2	0	1	13
*Stratum I**^1^***	78	45	15	0	5	0	2	3	1	19
*Area G*	23	23	9	1	2	3	2	1	0	5
**Total**	**235**	**160**	**33**	**4**	**20**	**5**	**9**	**11**	**5**	**68**

^1^ The pathological profile for this cemetery was examined but the results will be reported elsewhere because this cemetery is later (1700-1300 B.C.) and does not address the research question considered here.

Pathological lesions were recorded based on macroscopic observations of the human skeletal remains using standard methods. We noted the presence of proliferative and lytic lesions, as well as alterations to the morphology of the bones. Differential diagnosis was undertaken by comparing the presence and patterning of lesions with expectations from the paleopathology literature [119–128]. Because many individuals are fragmentary or incomplete, it was not always possible to examine the patterning of lesions beyond one or two elements. Thus our analysis is restricted to an evaluation of “periosteal reaction” in postcranial elements because this term does not connote a specific etiology, it refers to a response to inflammation, trauma, infection, or infectious disease in a single element [129]. Its lack of specificity makes it appropriate for use in fragmentary and incomplete remains. 

When the cranium was preserved, we evaluated evidence of maxillary infection and leprosy; lesions pathognomic of leprosy are primarily found in the craniofacial skeleton and evidence from the post-cranial elements is not required for a diagnosis. A diagnosis of leprosy was based on diagnostic criteria listed in Table 2. In one case, evidence for inflammation or infection (proliferative and destructive lesions on the external table of the cranial vault) was associated with cranial trauma. In a case like this, it is difficult to determine whether the periosteal reaction is solely related to the injury, or if infectious disease was also present. We erred on the side of caution and attributed these lesions to the category of “periosteal reaction”. Tuberculosis was only evaluated in cases where the individual had vertebrae and other post-crania available for study because vertebral changes are most diagnostic for tuberculosis.

**Table 2:** Specimen table for lesions associated with non-specific periosteal reactions, maxillary infection, leprosy, and tuberculosis.

	**Cemetery** R-37 (N=66)		**Cemetery H** (**stratum II**) (N=26)		**Area G** (N=23)	
	*Affected individuals *	*% affected*	*Affected individuals*	*% affected*	*Affected individuals*	*% affected*
**Non-specific infection**			**1**	**3.9**	**2**	**8.7**
Periostitis			H.698			
Osteomyelitis					II.32; I.S.11	
**Maxillary infection**	**1**	**1.5**	**2**	**7.7**	**1**	**4.4**
Periapical abscess	H.804		H.344		I.S.15	
AMTL	H.804		H.344; H.700a		I.S.15	
Alveolar resorption	H.804		H.700a		I.S.15	
Sinusitis			H.700a		I.S.15	
Porosity on the maxilla					I.S.15	
**Lesions consistent with leprosy**	**2**	**3.0**	**2**	**7.7**	**5**	**21.7**
Remodeling, pitting, vascular impressions, and/or new bone formation in the pyriform aperture	H.779		H.488		G.289; II.S.5; III.S.21	
Resorption of anterior nasal spine	H.779; H.820;		H.488		G.289; II.S.5; S#5; III.S.21	
Remodeling of the nasal margin	H.779; H.820		H.488		G.289; II.S.5; S#5; III.S.21	
Pitting and remodeling at the base of the nasal septum	H.779				G.289; II.S.5; III.S.21	
Exposure of the neurovascular channel in the pyriform aperture	H.779; H.820				G.289	
Porosity on the palatal surface and alveolar process of the maxilla	H.779; H.820				G.289; II.S.5; S#5; III.S.21	
Periodontosis	H.779; H.820		H.488		G.289; II.S.5; S#5; III.S.21	
Ante-mortem Tooth Loss (AMTL)	H.779; H.820		H.488		G.289; II.S.5; S#5	
Alveolar resorption to naso-palatine nerve	H.820		H.488			
Porosity on zygoma and orbital process of maxilla			H.488		II.S.5; III.S.21	
Pitting and grooves in the maxillary sinuses			H.488		G.289; S#5	
Periostitis on tibia/fibula					G.289	
Tarsal, metatarsal, and/or phalangeal osteomyelitis or sepsis					G.289; III.S.3/4	
Phalangeal concentric diaphyseal remodeling, palmar groove, cupping deformity, and/or interphalangeal ankylosis			H.696		G.289; III.S.3/4	
Changes to the ventral surface of naviculars and/or cunieforms					G.289; III.S.3/4	
Navicular squeezing					G.289	
**Lesions consistent with tuberculosis**			**2**	**7.7**		
Penetrating lytic lesions on the cranial vault < 2 cm in diameter			H.488; H.710			
Reactive bone formation on the zygoma and/or maxilla, necrosis of the mastoid process and/or petrous pyramid of the temporal			H.710			
Periosteal reaction or erosion of the occipital condyles, sphenoid body and/or greater wing						
Lupus vulgaris						
Reactive bone formation on ventral surface of vertebral centra			H.710			
Destructive focal lesions (cavitation) on vertebral centra			H.710			
Kyphosis of thoracic vertebrae						
Ankylosis of one or two affected vertebral bodies						
Periostitis or cavitation on ventral surface of ribs at vertebral end			H.722			
Lytic lesions on the manubrium, scapula, clavicle, humeral head, distal humerus, proximal radius or ulna, ilium, femoral head, femoral trochanters, femoral condyles, and/or tibial condyles			H.710; H.722			
Cavitation and/or sequestrum on the long bone shafts			H.710			
Lesions on carpals and/or tarsals			H.710			
**Total affected**	**3**	**4.5**	**7**	**26.9**	**8**	**34.8**

## Results

In our examination of the Harappan skeletons, we found evidence for non-specific periosteal reactions, maxillary infections, and individuals that demonstrate a pattern of lesions consistent with leprosy and/or tuberculosis (Table 2). Because the skeletal collection is fragmentary and many individuals are poorly preserved, many individuals were diagnosed with non-specific infections or maxillary infection when they might in fact have had an infectious disease but because the majority of the skeleton is not present a more detailed diagnosis is not possible [130]. Descriptions and images are provided in the following sections.

A summary of the number of individuals affected from each cemetery is provided in Figure 2. Prevalence of pathological conditions was calculated based on the proportion of individuals affected from each cemetery. For Cemetery R-37, 90 additional individuals were excavated from cemetery R-37 during the 1986-89 HARP project [52]. Those remains are stored elsewhere and were not available in the present analysis so they were not included in the proportion of affected individuals reported in the results section, Figure 2, or Table 2. As those individuals were studied previously for evidence of pathology, those prior results were included in the conclusions and discussion. 

### Non-specific periosteal reactions and infection related to trauma

One individual demonstrated evidence of a non-specific periosteal reaction, an adult (H.698) from stratum 2 of Cemetery H (3.9%). Individual H.698 had periostitis on a fragment of her tibial diaphysis. Because the tibia is fragmentary and the antimere is not present, this was recorded at non-specific periosteal reaction. There is no evidence of trauma, however it also cannot be ruled out as this individual also suffered from four violent injuries to her cranial vault [1]. 

**Figure 3:**
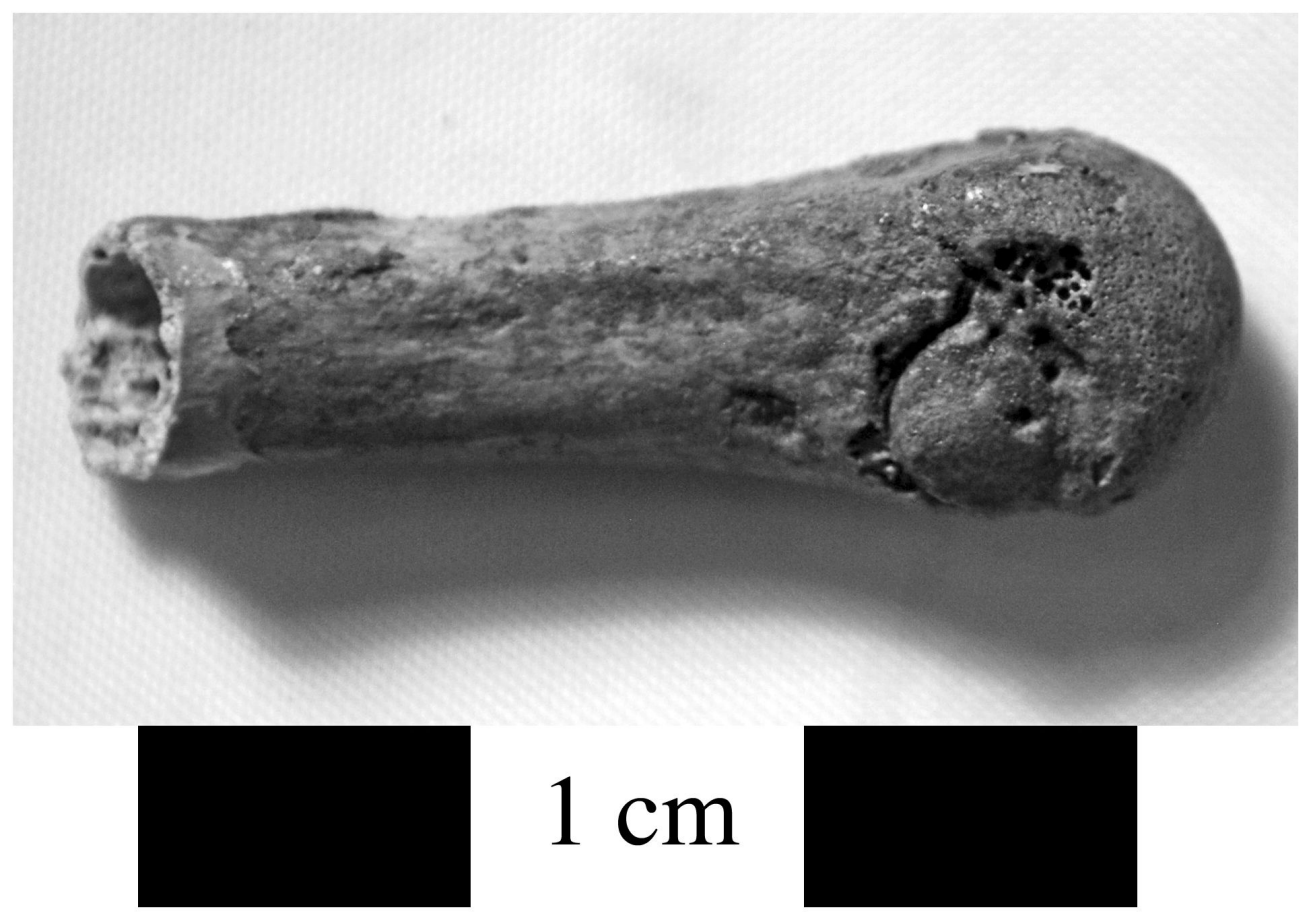
Infection in a metacarpal fragment from individual G.II.32 probably related to a local traumatic injury (lateral view).

**Figure 4:**
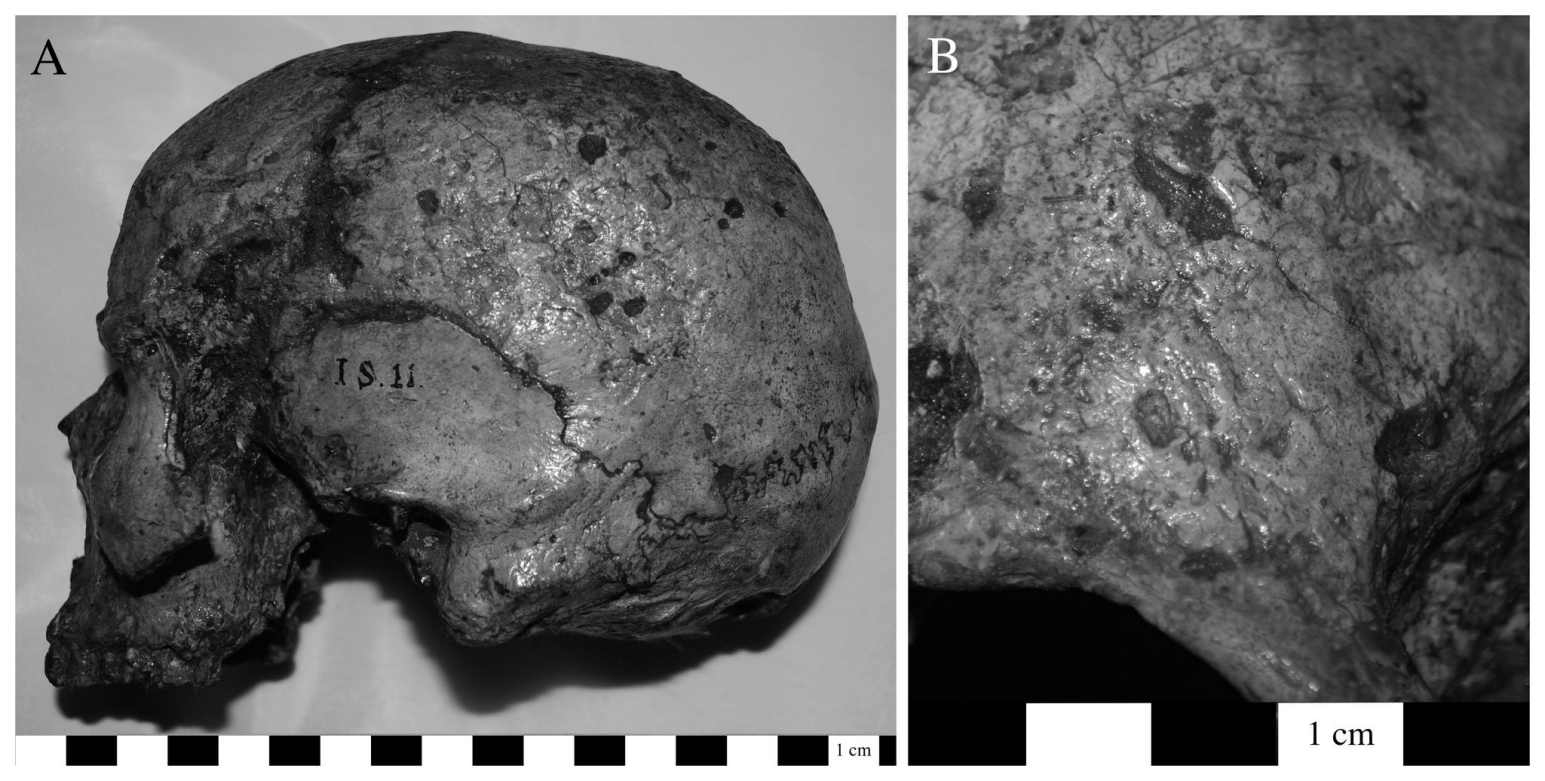
Lesions on the cranial vault of a male skull, I.S.11. This individual also has an injury (sharp blunt force trauma) on the frontal bone. The cranium is isolated and thus the etiology of these lesions is unclear (a: left lateral view). A close-up image demonstrates the destructive and proliferative character of these lesions (b: right frontal bone).

Evidence for infection related to trauma was found on two individuals from Area G (8.7%), one of which (G.II.32) consisted solely of an isolated metacarpal fragment with a sequestrum and involucrum (~5 mm) on the palmar surface of the head and a cloaca that has formed along the proximal margin (Figure 3). There is extracortical bone formation on the metacarpal head. There are no other remains clearly associated with this bone so the etiology of this lesion is unknown, although it appears to be a localized infection that probably resulted from an injury. Individual I.S.11 suffered from sharp blunt force trauma to the left frontal bone near glabella [1]. There is evidence for proliferative and destructive lesions on the external table of cortical bone of the left frontal and parietal (Figures 4a and 4b). We classified this as non-specific infection given that the cranium is the only skeletal element securely associated with this individual, and given the injury to the left frontal, which also demonstrates reactive bone formation along its margins. 

### Maxillary Sinus Infection

Evidence for maxillary sinus infection was found in one individual from Cemetery R-37 (H.804), one individual from Area G (G.I.S.15), and two individuals from Cemetery H stratum 2 (H.344 and H.700a). H. 804 had a complete splanchnocranium but most of the neurocranium is missing (except for the right temporal, sphenoid, and the anterior half of the right parietal). The post-crania were missing. This individual is not very well preserved but there are large periapical abscesses at the right and left lateral incisor and canine. The left lateral incisor was lost antemortem and the alveolus is completely resorbed. A severe level of attrition affects the right canine and the pulp chamber was exposed. There is porosity on the right frontal squama above the right eminence (a circular lesion approximately 2 cm in diameter). 

**Figure 5:**
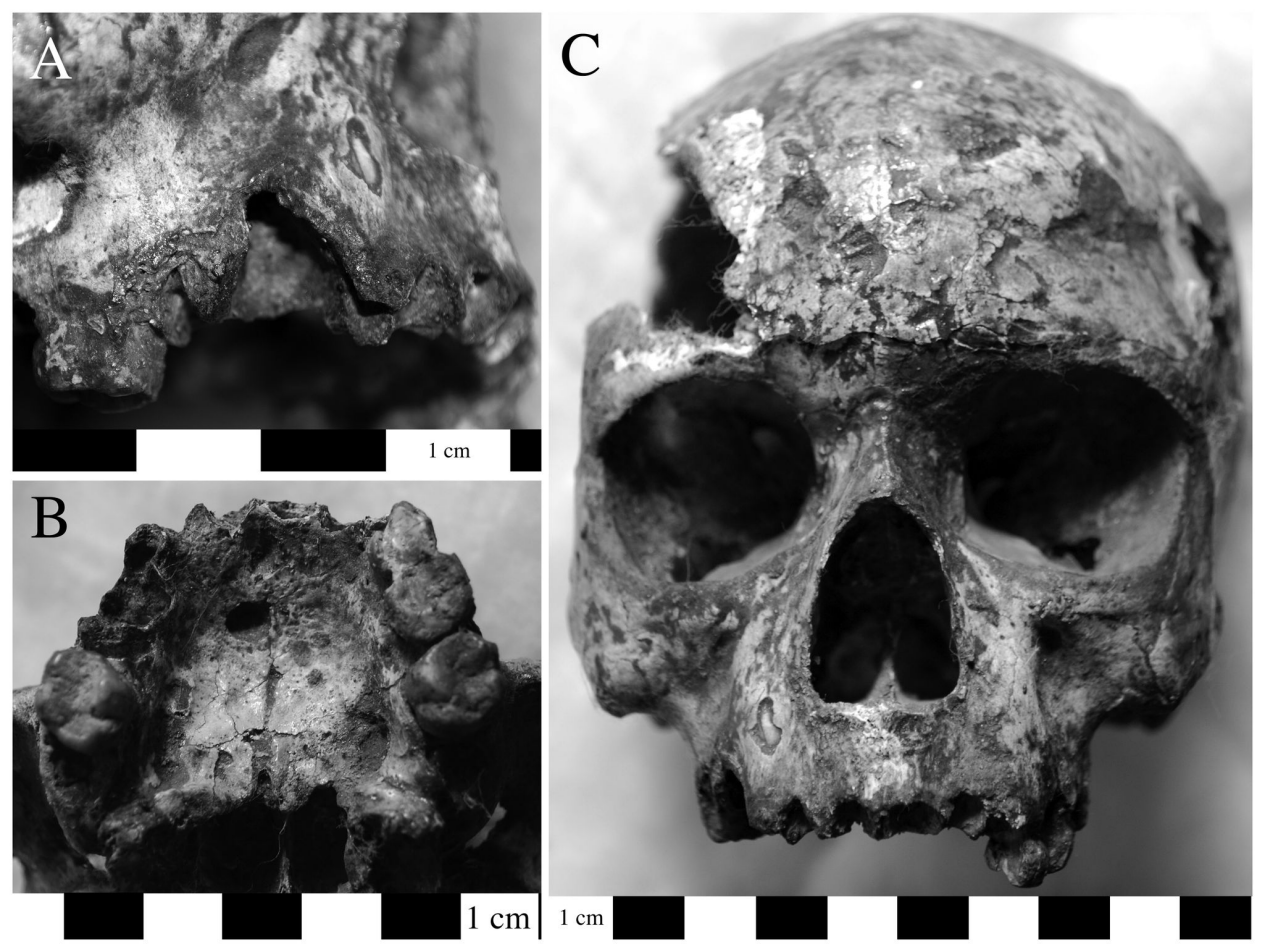
Evidence for maxillary infection in individual G.I.S.15. The lesions included porosity, alveolar resorption, abscessing at the right canine and third premolar, and antemortem tooth loss (a = right ventral view). This individual also had inflammatory changes to the palatine process of the maxilla leading to localized bone destruction and perforation (b = inferior view of palate). There is evidence for porosity and inflammation at the inferior margin of the pyriform aperture, porosity and deformation of the infraorbital foramen caused by infection of the left maxillary sinus (c: ventral view).

**Figure 6:**
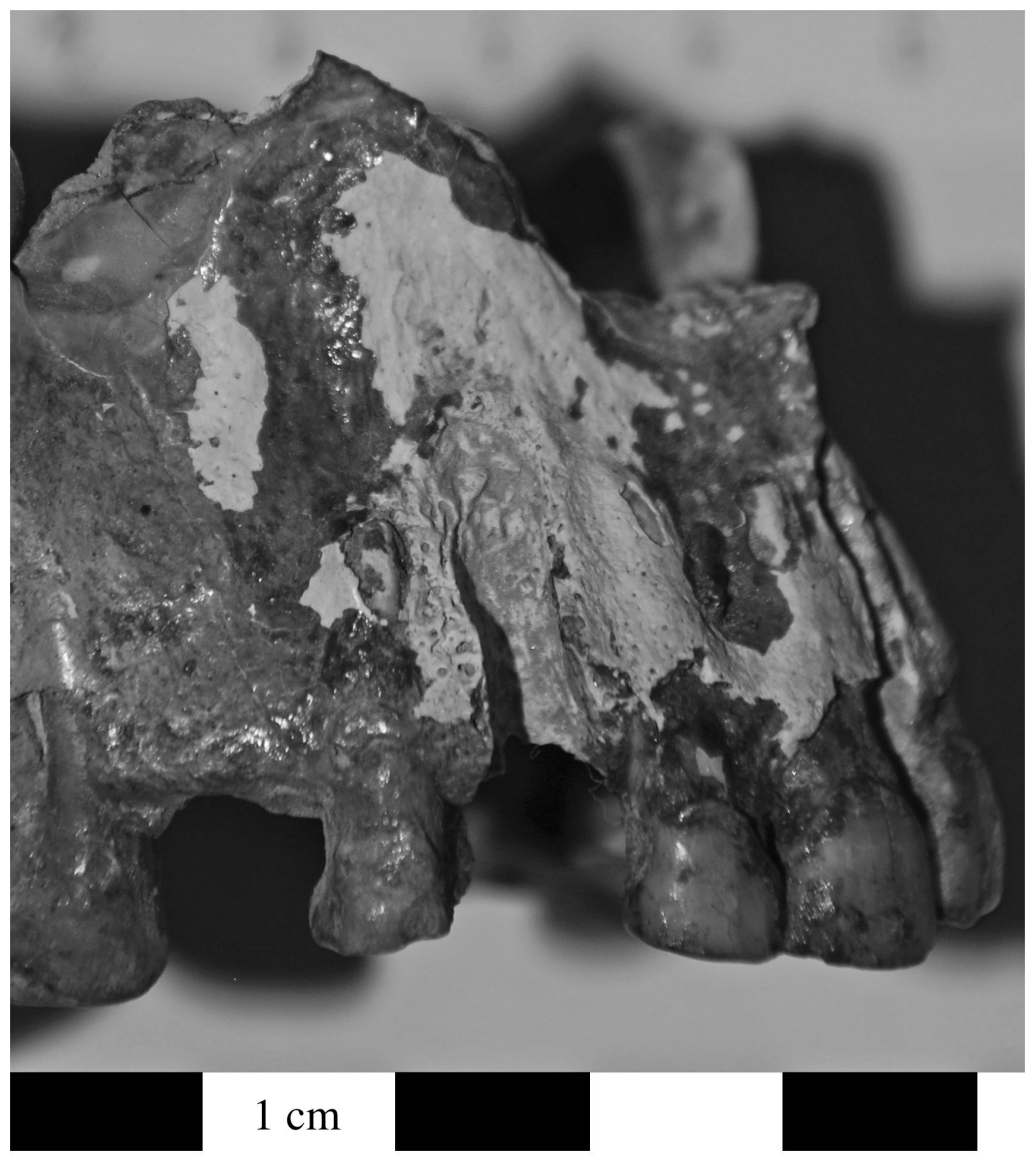
Porosity and alveolar resorption at the maxillary canine of individual H.700a (view of the right maxilla).

Individual G.I.S.15 from Area G demonstrated evidence of a severe maxillary infection. There is a large alveolar abscess on the left maxilla where the canine and third premolar were lost antemortem (Figure 5a). The right lateral incisor was lost antemortem and the alveolus was actively remodeling. The bony palate is porous and an opening behind the left central incisor has reactive bone margins and opens to an abscess in the anterior alveoli (Figure 5b). There is porosity on the alveolar bone of the maxilla, remodeling along the inferior margin of the pyriform aperture, including at the nasal spine, and porosity at the inferior orbital margin above an enlarged infraorbital foramen on the left maxilla (Figure 5c). Individual H.344 had an alveolar abscess at the left maxillary canine and third premolar. Individual H.700a lost the left maxillary canine antemortem; the ventral wall of the alveolus has been resorbed and the exposed surfaces are actively remodeling (Figure 6). The left sinus is exposed and the surfaces are porous, with reactive bone formation.

### Leprosy

**Figure 7:**
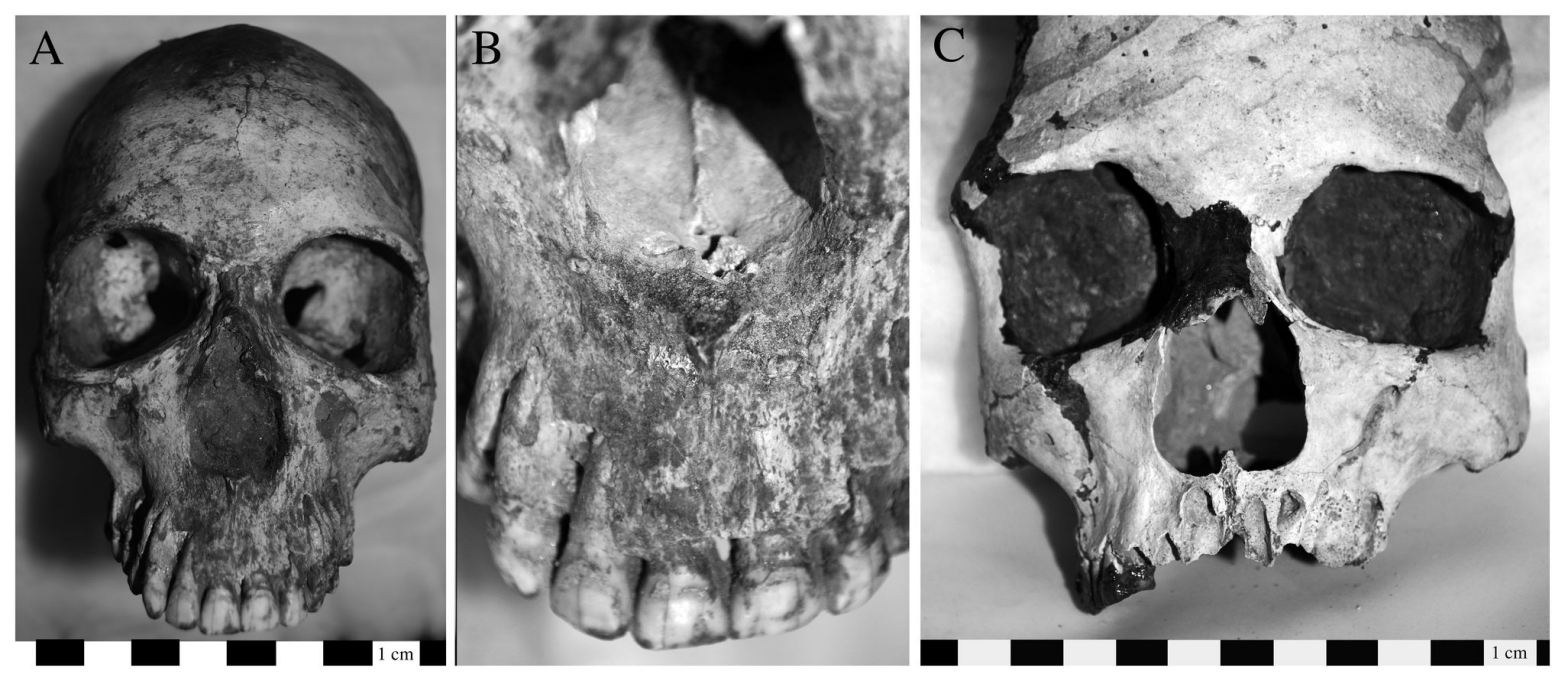
Severe rhinomaxillary infection is consistent with a diagnosis of leprosy. These lesions include resorption of the anterior nasal spine, changes to the inferior nasal margin, and remodeling of the nasal septum in individual H.779 (a: ventral view of cranium; b:superior view of pyriform aperture and maxilla). Among other changes indicative of leprosy, individual H.306a demonstrates recession of the anterior alveolar bone to the level of the naso-palatine nerve, porosity along the margin of and remodeling of the pyriform aperture, porosity on the bony palate, zygoma, nasal and orbital processes of the maxilla (b: ventral view).

There were two individuals from Cemetery R-37 (H.779 and H.820), a minimum number of five individuals from Area G (G.II.S.5, G.S.No.5, G.III.S.3/4, G.III.S.21, and remains labeled G.289), two individuals from Cemetery H stratum 2 (H.488, H.696) that demonstrated lesions consistent with a diagnosis of leprosy (Table 2). A diagnosis of leprosy was confined to individuals that demonstrated lesions related to severe rhino-maxillary infection: remodeling of the pyriform aperture is demonstrated by G.II.S.5 including resorption of the anterior nasal spine, changes to the anterior nasal margin, remodeling of the nasal septum, and exposure of the neurovascular channel (Figure 7a and b). Other changes associated with leprosy include resorption of the dental alveoli of the anterior dentition up to the level of the naso-palatine nerve, remodeling of the pyriform aperture, porosity on the bony palate, zygoma, nasal and orbital processes of the maxilla in H.306a (Figure 7c). 

**Figure 8:**
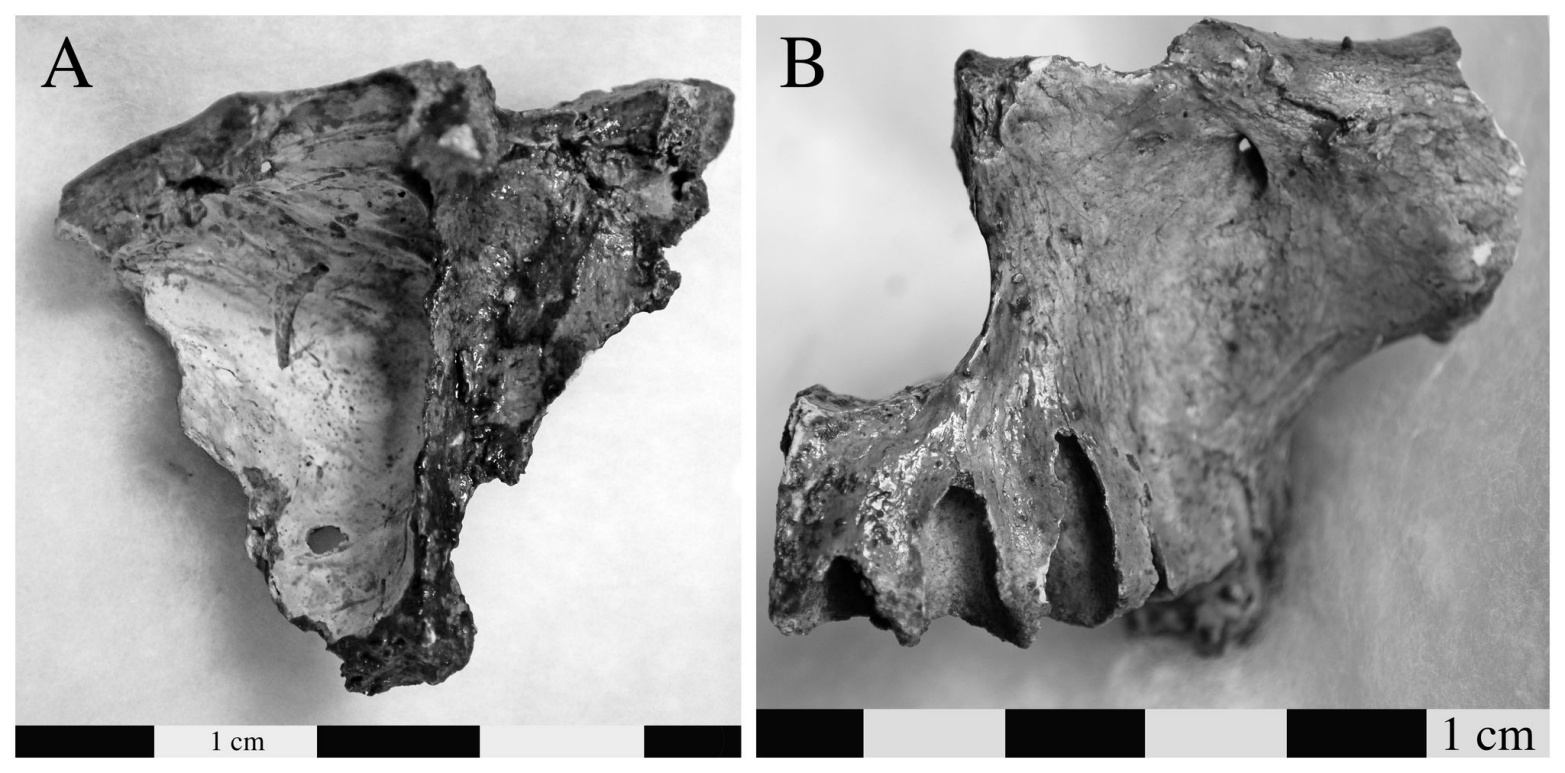
Individual G.289 was relatively complete for individuals in the Area G assemblage. Among other changes, this individual demonstrates porosity and reactive bone formation in the left maxillary sinus, as well as exposure of the neurovascular channel in the pyriform aperture (a: superior view of anterior and inferior surfaces of left sinus and floor of pyriform aperture). The anterior nasal spine has been remodeled; the alveolus for the left central incisor is resorbed; and there is porosity on the inferior margin of the left orbit (b: superior view of left maxilla).

These individuals also demonstrated reactive bone formation and porosity in the maxillary sinus, as demonstrated by a left maxilla labeled G.289 from Area G (Figure 8a). The floor of the pyriform aperture is covered with porous, reactive bone and there is remodeling along the anterior margin. There is also a perforation at the midline of the palate. New compact bone formation with a vascular pattern is present on the lateral margins of the nasal aperture. The anterior nasal spine has been remodeled, the alveolus for the left central incisor is resorbed, there is reactive bone along the alveolar margin, and there is porosity on the inferior margin of the left orbit (Figure 8b). The first and second molar are present but the anterior dentition was lost post-mortem. The alveolus for the third molar was actively remodeling; porosity is present on the interior surfaces of the alveolus and there is reactive bone formation along its alveolar margin. 

**Figure 9:**
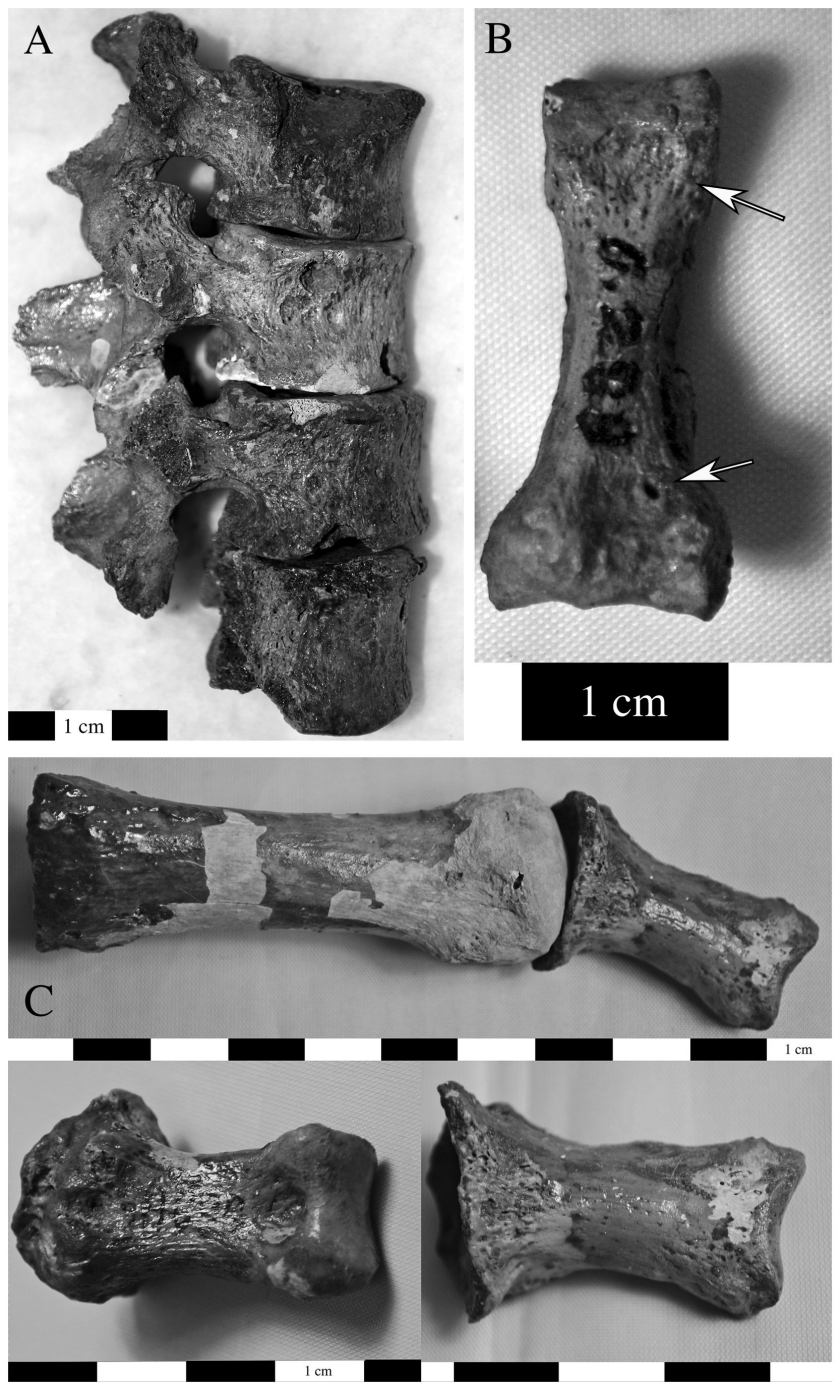
Individual G.289 also demonstrated postcranial changes, some of which are consistent with a diagnosis of leprosy. There is evidence of supperative osteomyelitis in the vertebral column, with focal lytic lesions on the ventral and left lateral surfaces of a thoracic centrum and osteosclerotic reaction, erosion of the anterior rim, and hypertrophic new bone formation on adjacent vertebral bodies (a: lateral view). Of other postcranial changes consistent with leprosy, there was a palmar groove and a small cloaca on an proximal manual phalanx (indicated by arrows on image b: palmar view); evidence for inflammation and/or infection causing bone overgrowth on the first pedal phalanx and metatarsal (c: top image is of the superior surface; superior and inferior view of the proximal phalanx are on the left and right side on the bottom row).

A first cervical vertebra, four lumbar vertebrae, a fibular shaft fragment, right and left navicular, left intermediate and lateral cuneiform, and left fourth metatarsal were also labeled G.289 but this label refers to the provenience of the remains and their association with the maxilla is not definite but is highly likely. Postcranial changes included evidence of supperative osteomyelitis in the vertebral column (G.289). The vertebral centra were affected by focal lytic lesions on the left side, osteosclerotic reaction, erosion of the anterior rim, and hypertrophic new bone formation (Figure 9a). Postcranial changes consistent with leprosy include periostitis on the fibula, porosity and a palmar groove on an intermediate manual phalanx (Figure 9b), reactive bone formation and osteomyelitis of tarsals, including the naviculars, navicular squeezing, periostosis and septic changes to the joint surfaces on the first metatarsal and associated phalanx (Figure 9c). 

### Tuberculosis

**Figure 10:**
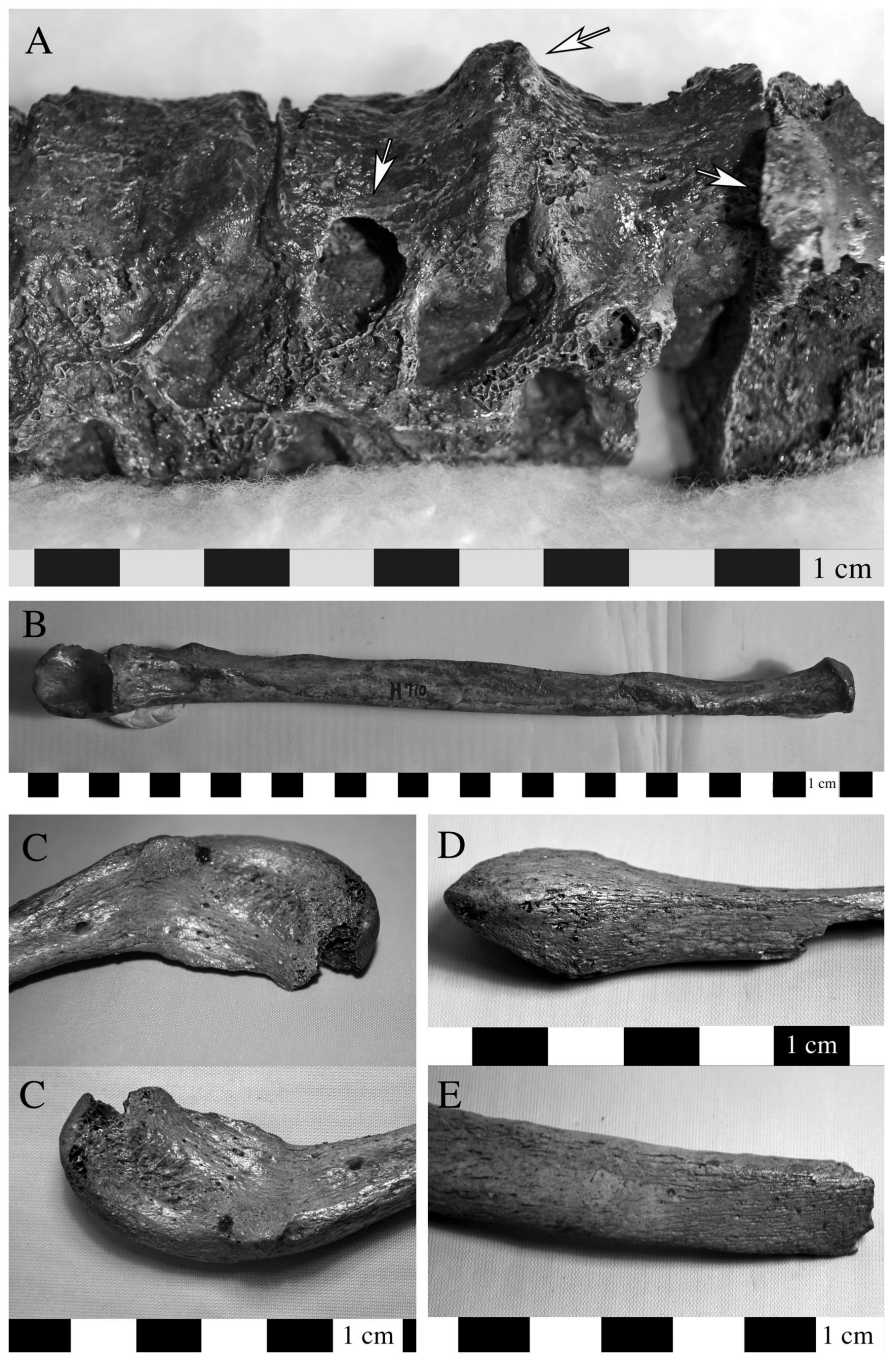
Two individuals from Cemetery H demonstrated cranial and postcranial lesions consistent with a diagnosis of tuberculosis. The postcranial lesions included a smooth walled cavitation consistent with evidence for a psoas abscess formation on the right side of the ninth thoracic vertebral centrum and associated reactive bone formation and ankylosis of the adjacent elements in H. 710 (a: lateral view, with arrows indicated abscess and reactive bone formation or ankylosis); osteomyelitis affected the distal third of the left ulna (b: ventral view). H.722 demonstrated evidence of inflammation and bone swelling in the acromial end of the right clavicle (c: inferior view), periostitis on the left distal fibula (d: lateral view), and inflammatory changes to the surface of a left rib at the angle (e: dorsal view).

 Evidence for skeletal lesions consistent with a diagnosis of tuberculosis was limited to two individuals (H.710 and H.722) from Cemetery H, stratum 2 (Table 2). The evidence from H.710 included reactive bone formation on the anterior centrum of the cervical vertebrae; vertebral changes consistent with psoas abscess formation in the thoracic vertebrae (cavitation on the right lateral centrum) associated with reactive bone formation and ankylosis of the adjacent elements (Figure 10a); abscess formation in the distal third of the left ulna (Figure 10b); porotic spongiosa on the femoral head; periostitis and reactive endosteal bone formation on the tibia; hallux valgus; periosteal reaction on the plantar surface near the head of the left first metatarsal; and bloated periosteum on the second metatarsal base. Individual H.722 was more fragmentary but demonstrated evidence for osteomyelitis in the acromial end of the right clavicle (Figure 10c); endosteal bone deposition on a fragment of left distal tibia (Figure 10d); periostitis on both the pleural and the dorsal surface of the ribs at the angle (Figure 10e); and reactive bone changes on the cuneiforms and naviculars, including navicular squeezing. 

**Figure 11:**
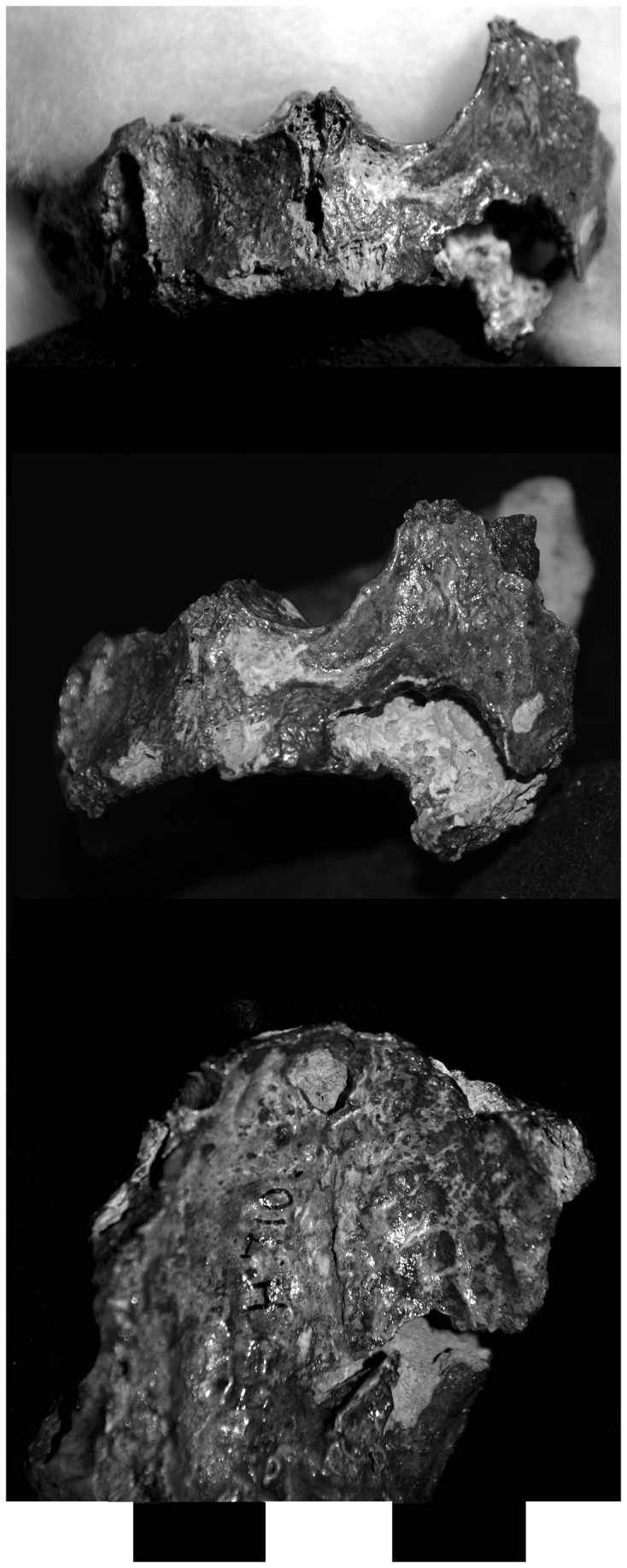
Changes to the maxilla in H. 710 that are consistent with the diagnosis of tuberculosis include abscess formation and destruction of the maxillary alveoli, periosteal reaction in the right maxillary sinus, destruction of the bony palate and initial changes leading to perforation at the midline of the palate (ventral view image on top, left lateral view in center, and inferior view of maxilla on the bottom).

One of these individuals (H.710) also had cranial remains present and demonstrated evidence of abscess formation and destruction of the maxillary alveoli; periosteal reaction in the right maxillary sinus; destruction of the bony palate and initial changes leading to perforation at the midline (Figure 11); and a healing lesion approximately 2 cm in diameter on the frontal squama. Because of the advanced state of healing it is difficult to determine whether this is related to infection or trauma; the latter explanation is preferred [1]. This individual also has a traumatic injury on the occipital bone [1]. 

## Discussion

We are broadly interested in understanding how human health was impacted by environmental and social changes in the past. Theoretically, challenges like urbanization, extensive culture contact, climate and socio-economic changes would exacerbate the risks for the spread of infectious diseases. Based on prior evidence for leprosy in the human population at the peripheral settlement of Balathal circa 2000 B.C. [82], and molecular evidence for long-standing interaction among MTBC lineages and human populations in South Asia [75,128], we hypothesized that there would be evidence for infectious diseases from the MTBC family in the skeletal populations from Harappa. Our results provide support for this hypothesis and from these data we infer that: 1) the presence of leprosy in the urban phase skeletal sample from cemetery R-37 demonstrates the disease was present at Harappa before the end of period IIIC (2030 Cal. B.C.) [108], 2) urbanization and culture contact likely facilitated the spread of MTBC lineages in Indus populations, 3) it is also likely that mycobacterial pathogens migrated between South and West Asia and Africa as part of the Third Millennium interaction sphere [76,81], and finally 4) the low incidence of infection with leprosy (3.0%) in the remains studied from this period suggests the disease was *relatively* uncommon in the urban phase, as was infection in general (4.5%).

Our second prediction was that the prevalence of infection and infectious disease will vary through time at Harappa, correlated with fluctuating levels of environmental, social, and economic stress. The combined evidence from mortuary treatment, traumatic injury, and infection indicate that the stresses of the post-urban period were accompanied by increasing levels of inter-personal violence and disease. Leprosy was present in a relatively small proportion of individuals during the urban period but the post-urban phase saw increasing risks for health and safety. By the Late Harappan phase, leprosy and tuberculosis affected 15.4% of the individuals buried at Cemetery H and leprosy affected 21.7% of the Area G specimens. Combined with our previous work demonstrating that the prevalence of violence also increased through time, affecting men, women, and children from Area G at an unequaled rate of 50% [1], we infer aspects of identity or behavior created greater vulnerability in some Harappan communities. 

This paper reports the results of an analysis of 115 skeletons from Harappa. The human remains are incomplete; thus if molecular techniques become possible in the future, these individuals would be good candidates for analysis. While our results do support the hypothesis that disease burden increased with climate and social changes in the Late Harappan phase, the present analysis only concerns paleopathological indicators for individuals who were buried, preserved, and excavated at Harappa. The skeletal sample does not reflect the percent individuals affected in the living population. While this is obviously only a small fraction of the living population size, it is also the largest sample of skeletons that has been studied from an Indus city thus far. Our data include individuals from three different burial areas at Harappa, spanning a period of 700 years, making the BHaRaT (Bioarchaeology of Harappa, Research and Training) project the most comprehensive survey of skeletal pathology from an Indus city to date. While the paleoepidemiological profiles are not directly representative of the specific risks of violence and infection should the entire living population be considered, these data are useful for making comparisons of the relative risks among burial communities through time and the human remains do inform us about social processes, as they were inscribed on the skeletons preserved at the site. 

An alternative explanation for these data is that burial traditions changed through time and individuals affected by disease were increasingly likely through time to be buried, as opposed to some other means of disposal. However, an examination of differences among the skeletal assemblages demonstrates that the presence of disease alone is insufficient to explain mortuary treatment, including whether an individual would be included or excluded from the cemetery. Evidence for infection was present in small frequency in Cemetery R-37 and in Cemetery H Stratum II. In the latter sample, 26.9% of the skeletons we analyzed demonstrated evidence of pathological lesions consistent with a diagnosis of leprosy. These individuals were accorded the same burial treatment as skeletally unaffected individuals. Thus we argue that disease alone was insufficient to warrant exclusion from or inclusion in the cemetery population. 

Twenty-three individuals were accorded very different mortuary treatment at Harappa. They were interred at Area G, southeast of the city. This area consisted of some poorly preserved architectural remains that were interpreted as small dwellings, alongside a pit of 20 male, female and immature crania and isolated postcranial remains [7]. The crania piled in this trench demonstrated the highest prevalence of infection and infectious disease of any skeletal assemblage at Harappa (34.8% of 23 individuals). Our previous research also demonstrated a high risk for violent injury among these individuals [1]. These results demonstrate that individuals who were excluded from the cemetery were also the most vulnerable and at the highest risk for infection, infectious diseases, and interpersonal violence.

Stigma has often accompanied leprosy in the historic period [131], and the presence of lepers outside of city walls is almost a cliché in the paleopathology literature. At Harappa, the mortuary context at Area G could be interpreted as evidence for stigma. Epidemiologically, the greatest risk for violence and disease occurred in those individuals who were excluded from the cemetery. Leprosy and traumatic injury co-occur at Area G. However, traumatic injuries, leprosy and tuberculosis also co-occur in the cemetery populations, suggesting that individuals in Area G were not excluded from the cemetery because of disease. A more parsimonious interpretation is that these individuals were more vulnerable to violence and disease because of other aspects of their identity—sex, age, social status, community, and perhaps behavior—that also led to exclusion from the cemetery. It is uncertain whether the people interred here were marginalized because they suffered from an infectious disease, or whether they suffered from a higher prevalence of violence and disease because they were marginalized, impoverished, and stigmatized for some other reason. We favor the latter explanation but deeper analysis of mortuary treatment may help to address some of these questions and is planned for a future study.

 Future studies must also involve a deeper investigation of co-occurrence of trauma and infection; co-occurrence of infectious organisms in individual skeletons; and, an analysis of other indicators of developmental stress and growth disruption, which will clarify whether affected individuals demonstrate evidence for chronic deprivation, or stress markers earlier in the life course. More specific information about stigma may also be obtained by analysis of additional skeletal populations from this time period, particularly if contemporary excavations are focused on areas outside the city walls.

### Conclusion

Currently we face global climate changes of a projected magnitude unprecedented in the Holocene epoch [132]. We are already experiencing increases in mean atmospheric carbon, land surface temperatures, and sea levels. Perhaps more significantly, as the mean values increase, the weather is becoming more variable, precipitation patterns are changing, and we are experiencing more frequent severe storms [133]. Drought, flooding, and other natural disasters are expected to increase in frequency; uncertainty and unpredictability may eventually make basic goals like subsistence more difficult. 

Human security literature predicts that in the current context, when a proportion of the population in a complex society (or “state”) is disadvantage by socio-cultural, historical, economic, and political processes, it is these communities who face disproportionate effects from food shortage, reduced access to raw materials, sanitation issues, conflict, and disease [63,85,86,91,92,134–137]. Historically, even relatively minor climatic or environmental changes have led to increasing levels of violence, starvation, epidemics, and/or biodemographic changes in human populations [137–139]. While it is not always the case, resource scarcity, environmental uncertainty, and declining agricultural production in the face of continued population growth have resulted in starvation, violence, and/or emigration many times in prehistory as well (see for example, 140–147). 

The human skeletal remains from Harappa examined here demonstrate this association in South Asian prehistory. However, our results should not be misinterpreted as confirming an adaptationist perspective that elevated levels of competition for scarce agro-ecological resources and increased levels of migration during periods of climate change necessarily lead to increases in conflict, interpersonal violence, and negative impacts on human health. Despite claims in popular books [148,149], the fate of human societies is not determined by one or two extrinsic factors. Human communities in the past had a variety of responses to climate change, resource scarcity, and conflict [68,150–150–153]. Biocultural approaches to human prehistory have repeatedly demonstrated that is the social world that determines the meaning of and responses to environmental stressors, and it is the social structure and human agency that determine who will be affected, how, and to what degree [154–157].

Human populations in semi-arid regions of the world, including South Asia, currently face disproportionate impacts from global climate change. The evidence from Harappa offers insights into how such changes impact human societies. South Asians have historically had diverse responses to climate change [144]. At Harappa, the same forces that characterized the process of urbanization and social stratification probably ultimately shaped the collapse. Climate change and exchange relationships may have enhanced immigration to the city of Harappa after 2200 B.C. and the archaeological record suggests that Indus people successfully managed the pressures of climate change, migration, and rapid population growth for centuries prior to 2000 B.C. However, climate change and massive population growth had an increasingly negative impact on the city through time, creating an environmentally, demographically induced pressure point [158]. Centuries of hydro-ecological stress, agro-economic problems, increasingly frequent interruptions of the exchange network, and deterioration of conditions in the city eventually led to massive depopulation around 1900 B.C. Those who remained suffered from increasing rates of interpersonal violence and infection, particularly in marginalized communities who received little protection from a weak, decentralized society. The evidence from Harappa confirms the importance of both social and biological challenges in shaping the fate of human populations dealing with rapid population growth and environmental degradation. 

Future archaeological research should further examine the possibility that Harappa offers an historical example of how climate and social changes in a weak and deteriorating “state”, or “pre-state” level, complex society can disproportionately impact disadvantaged, marginalized, or vulnerable communities. One question that arises is whether the evidence from Harappa is principally demonstrative of biosocial processes at work in this one city, or whether social differentiation and status differences typified the process of urbanization in other Indus communities. In other words, how typical is this pattern of the Indus civilization as a whole? Archaeologists have long known there was some evidence for interpersonal violence in some of the assemblages from Indus Civilization cities [105,113,159–165]. However, evidence for trauma and pathology has been controversial, much debated, and sometimes minimized as interpretations have been limited to discussions about Aryan Invasion mythology and there has been little attempt to understand how these lesions relate to the intrinsic social forces that were at work in the ancient populations [6,113,166]. Pending further investigation of skeletal material from additional sites like Mohenjo Daro, Lothal, Chanhu Daro, and Kalibangan, it is safe to say that Harappa was not an isolated, peripheral settlement on the fringes of Indus society. It was one of the largest regional centers in the Indus Valley and has often been used as a primary source of information about the Indus Civilization. There is no evidence that Harappan society was markedly different from that of other urban centers, that it was special or deviant in some way, but this question must be examined through a larger, regional survey of paleopathological indicators. Social forces at work at Harappa are to a degree, representative of the larger processes at work in the Indus Civilization as a whole. Additional research on mortuary treatment, trauma, and pathology in other Indus cities is crucial to understanding how short-term strategies for coping with environmental, social and economic changes led to long-term consequences for human populations in South Asian prehistory.
